# Comparison of 22G standard and Franseen needles in endoscopic ultrasound-guided fine-needle aspiration for diagnosing pancreatic mass lesions: Study protocol for a controlled trial

**DOI:** 10.1186/s13063-019-3946-7

**Published:** 2019-12-30

**Authors:** Masahiro Itonaga, Satoru Yasukawa, Toshio Shimokawa, Mamoru Takenaka, Nobuyasu Fukutake, Takeshi Ogura, Junichi Sakagami, Hideyuki Shiomi, Yasushi Okura, Osamu Inatomi, Hisakazu Matsumoto, Akira Kurita, Azumi Suzuki, Kiyohito Tanaka, Masayuki Kitano

**Affiliations:** 1 Second Department of Internal Medicine, Wakayama Medical University, 811-1 Kimiidera, Wakayama City, Wakayama 641-0012 Japan; 2 Department of Pathology, Kyoto Second Red Cross Hospital, Kyoto, Japan; 3 Department of Surgical Pathology, Kyoto Prefectural University of Medicine, Kyoto, Japan; 4 Clinical Study Support Center, Wakayama Medical University, Wakayama, Japan; 5 Department of Gastroenterology and Hepatology, Kindai University Faculty of Medicine, Osaka, Japan; 6 Department of Hepatobiliary and Pancreatic Oncology, Osaka International Cancer Institute, Osaka, Japan; 7 The Second Department of Internal Medicine, Osaka Medical College, Takatsuki, Japan; 8 Department of Medicine, Division of Gastroenterology and Hepatology, Kyoto Prefectural University of Medicine, Kyoto, Japan; 9 Division of Gastroenterology, Department of Internal Medicine, Kobe University Graduate School of Medicine, Kobe, Japan; 10 Department of Microbiology and Infectious Diseases, Nara Medical University, Nara, Japan; 11 Division of Gastroenterology, Shiga University of Medical Science, Otsu, Shiga Japan; 12 Department of Gastroenterology and Hepatology, Japanese Red Cross Society of Wakayama Medical Center, Wakayama, Japan; 13 Division of Gastroenterology and Hepatology, Digestive Disease Center, Kitano Hospital, Tazuke Kofukai Medical Research Institute, Osaka, Japan; 14 Department of Gastroenterology, Kyoto Second Red Cross Hospital, Kyoto, Japan

**Keywords:** EUS-FNA, Puncture needle, Franseen needle, Pancreatic mass, Malignancy, Diagnostic yield

## Abstract

**Background:**

Endoscopic ultrasound-guided fine-needle aspiration (EUS-FNA) was developed with the aim of further improving the diagnostic performance of endoscopic ultrasound. Although novel puncture needles have been specifically designed for collecting sufficient tissue specimens, clinical studies have indicated no clear difference in diagnostic performance between these novel needles and conventional puncture needles. Recently, a needle with Franseen geometry was developed specifically for EUS-FNA biopsy. Due to the characteristic shape of its tip, the Franseen needle is expected to be effective for scraping tissues, thus potentially increasing the diagnostic accuracy of EUS-FNA biopsy. We plan to carry out a prospective, multicenter, open-labeled, controlled trial to compare conventional and Franseen needles in terms of the diagnostic accuracy of EUS-FNA for evaluating the malignancy of pancreatic mass lesions.

**Methods/design:**

The study will enroll 520 patients with pancreatic mass managed at any of 21 participating endoscopic centers. Lesion samples obtained using 22G conventional and Franseen needles will be assessed to compare the efficacy and safety of these two types of needles in EUS-FNA for evaluating the malignancy of mass lesions in the pancreas. Tissue samples will be fixed in formalin and processed for histologic evaluation. For the purpose of this study, only samples obtained with the first needle pass will be used for comparing the: (i) accuracy of the malignancy diagnosis, (ii) sensitivity and specificity for the malignancy diagnosis, (iii) procedure completion rate, (iv) sample cellularity, and (v) incidence of complications. Patient enrollment begins on July 17, 2018.

**Discussion:**

The outcomes of this study may provide insight into the optimal needle choice for evaluating the malignancy of pancreatic solid lesions, thus aiding in the development of practice guidelines for pancreatic diseases.

**Trial registration:**

University Hospital Medical Information Network Clinical Trials Registry (UMIN-CTR), UMIN000030634. Registered on 29 December 2017.

http://www.umin.ac.jp/

Version number: 01.2017.12.28.

## Background

Endoscopic ultrasound (EUS) is recognized as an effective tool for detecting, diagnosing, and T-staging small lesions in the vicinity of the gastrointestinal tract [1]. However, EUS has limited use in distinguishing between benign and malignant lesions. EUS-guided fine-needle aspiration (EUS-FNA) was developed with the aim of further improving the diagnostic performance of EUS. The use of EUS-FNA has become widespread because this technique provides a great deal of information that can be used to determine which treatment strategy is best suited in each case, depending on the disease stage and other properties. EUS-FNA is currently used especially for the diagnosis of pancreatic tumors. To date, clinical data have shown that EUS-FNA contributes to the treatment results in patients with pancreatic cancer [2]. In patients with solid pancreatic masses, EUS-FNA has a diagnostic sensitivity of 54–96%, specificity of 96–98%, and accuracy of 83–95% [3–6]. Cytopathology plays an important role in improving the diagnostic yield. While cytology can differentiate between benign and malignant lesions, histology evaluation must be performed in order to establish the lesion subtype and pathogenesis. It is thus necessary to collect sufficient tissue specimens to facilitate accurate histological evaluation. In this context, it is important to use puncture needles suitable for the particular features and location of the evaluated lesion [7].

Puncture needles specifically designed to collect sufficient tissue specimens have been developed, but clinical studies have not reported any clear differences in diagnostic performance for different needles [8–10]. Recently, a needle with Franseen geometry (Acquire™; Boston Scientific Corporation, Natick, MA, USA) was designed for EUS-FNA biopsy. Due to the characteristic shape of its tip, the Franseen needle is expected to be effective for scraping tissues, allowing collection of larger specimens, and thus potentially facilitating the diagnosis of lesions that are typically difficult to diagnose based on cytology alone, such as autoimmune pancreatitis and pancreatic endocrine tumors [11]. Therefore, the impact of the needle type (conventional or Franseen) on the diagnostic yield warrants further study. We plan to carry out a controlled trial to compare the diagnostic yield of 22G conventional needles (Expect™; Boston Scientific Corporation, Natick, MA, USA) and of the newly developed Franseen needles (Acquire™) used for EUS-FNA sampling of pancreatic mass lesions.

## Methods/design

### Ethics approval and patient consent

The study was approved by the Wakayama Medical University Ethics Committee (Institutional Review Board number 2135). Informed consent will be obtained from all patients. The trial was registered with the University Hospital Medical Information Network (trial registration number UMIN000030634). This protocol was prepared in conformance with the Standard Protocol Items: Recommendations for Interventional Trials (SPIRIT) guidelines (Additional file 1).

### Study aims and design

**Fig. 1:**
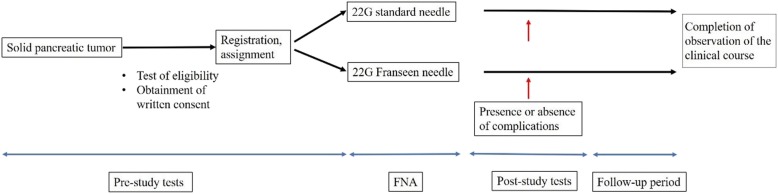
Flowchart illustrating the recruitment, allocation, and follow-up process

The major objective of this study is to determine whether the Franseen needle is superior to the conventional needle for accurate diagnosis of malignancy in patients with pancreatic mass. This multicenter controlled study will compare 22G conventional and Franseen needles in terms of the efficacy and safety of EUS-FNA for evaluating the malignancy of mass lesions in the pancreas. As this comparative study mainly aims to assess the specimens obtained with the first puncture, no special protocol will be established regarding the technique for the second or subsequent punctures. Outcomes of the second and subsequent EUS-FNA procedures will be used to make the final diagnosis of the pancreatic mass for patients with non-resection. The flowchart in Fig. 1 illustrates the recruitment, allocation, and follow-up process.

### Outcomes

The primary endpoint in this study will be diagnostic accuracy for malignancy, whereas secondary endpoints will include (i) sensitivity and specificity for diagnosis of malignancy, (ii) procedure completion rate, (iii) sample cellularity, and (iv) incidence of complications.

### Analysis target group

In the effectiveness analysis population, the full analysis set (FAS) is the main analysis and the per-protocol set (PPS) analysis is performed as a reference. In the safety analysis target population, all test samples are analyzed.

The FAS is defined as all registered cases excluding those who do not meet the eligibility criteria of this study after registration. The safety analysis target population is defined as cases who received part or all of the protocol treatments among all registered cases. The PPS is defined as cases excluding those meeting the following criteria from the FAS:

1. Inability to evaluate efficacy due to inadequate observation
2. Critical deviation violating the specifications of the protocol

### Major analysis and judgment criteria

The primary objective of this study is to determine whether the test diagnostic device (22G Acquire™ needle) is superior to the conventional diagnostic device (22G Expect™ needle) for accurate diagnosis of malignancy. If the rate of correct diagnosis is significantly higher in the Franseen needle group than in the conventional needle group, it will be concluded that the test diagnostic device (22G Acquire™ needle) is a more promising diagnostic device. If the difference is not significant, it will be concluded that the Franseen needle is not a superior diagnostic device compared with the conventional needle. The main analysis will use the Mantel-Haenszel test because adjustment factors other than the site are used for stratification in the FAS. When comparing the two groups in terms of the rate of correct diagnosis, the difference will be considered significant if the *p* value in the Mantel-Haenszel test is below the significance level taken into consideration in the power analysis (a two-sided significance level of α = 0.05). The Clopper-Pearson exact test will be used to estimate the 95% confidence interval of accuracy for the diagnosis of malignancy in each group. In addition, logistic regression analysis with adjustment for covariates (other than site) will be used to calculate the odds ratio (with 95% Wald confidence interval) for the outcome associated with using the Franseen needle versus using the conventional needle.

### Secondary efficacy analysis

#### Sensitivity and specificity for the diagnosis of malignancy

Sensitivity analysis is restricted to the subjects with malignancy and specificity analysis is restricted to the subjects without malignancy.

Sensitivity and specificity (with the Clopper-Pearson exact 95% confidence interval) will be estimated for each group in the FAS. The chi-square test will also be conducted, and the odds ratio (with 95% confidence interval) will be calculated to evaluate the between-group differences in sensitivity and specificity. The adjusted odds ratio (with 95% confidence interval) will be calculated using logistic regression analysis including the assignment factors.

#### Procedure completion rate

Procedure completion rate (with the Clopper-Pearson exact 95% confidence interval) will be estimated for each group in the FAS. The chi-square test will also be conducted and the odds ratio (with 95% confidence interval) will be calculated to evaluate the between-group differences in procedure completion rates. The adjusted odds ratio (with 95% confidence interval) will be calculated using logistic regression analysis including the assignment factors.

#### Sample cellularity

Sample cellularity will be tabulated by grade for each group in the FAS. The chi-square test will also be conducted to evaluate the between-group difference in tissue sampling rate. The adjusted odds ratio (with 95% confidence interval) will also be calculated using a proportional odds model including the assignment factors.

#### Incidence of complications

The incidence of complications (with the Clopper-Pearson exact 95% confidence interval) will be estimated for each group in the FAS. The chi-square test will also be conducted and the odds ratio (with 95% confidence interval) will be calculated to evaluate the between-group differences in the incidence of complications. The adjusted odds ratio (with 95% confidence interval) will be calculated using logistic regression analysis including the assignment factors.

### Setting

A total of 520 patients will be recruited from 21 endoscopic centers in Japan.

Registration will be performed at each participating center following local ethics committee approval of the study protocol and of the informed consent documentation and forms.

### Eligibility criteria

The following inclusion criteria will be applied: (i) age ≥ 20 years; (ii) pancreatic mass lesion detected on diagnostic imaging; (iii) performance status ≤ 2; (iv) indication for histological evaluation to predict the clinical course and to select treatment methods; (v) written consent obtained following adequate explanation of the study aims, design, and procedures.

The following exclusion criteria will be applied: (i) blood vessel or other tumors located between the lumen of the gastrointestinal tract and the target lesion; (ii) bleeding tendency, defined as an international normalized ratio of the prothrombin time > 1.5 or platelet counts < 50,000 cells/μl; (iii) any condition expected to hinder endoscope insertion; (iv) gastrointestinal reconstruction after gastrectomy; (v) serious complication involving another organ; (vi) known diffuse autoimmune pancreatitis; (vii) any other condition or situation determined by a study investigator to represent reason for ineligibility.

### Registration of candidates

At each participating center, the on-site study investigators will obtain informed consent from the candidates and use an electronic data capture system to confirm that the candidates meet the eligibility criteria (i.e., the candidates meet all the inclusion criteria and none of the exclusion criteria), input the necessary information, and register the candidates with the registration secretary. After confirming that the candidate meets the criteria, a registration number will be issued, and the registration will be considered complete. If any required input data are missing or the candidate does not meet the eligibility criteria, no registration number will be issued, and registration will be deemed incomplete. For each candidate, the date of registration is defined as the date when the registration number was issued by the registration secretariat via the electronic data capture system.

### Treatment allocation and blinding

Each subject will be assigned to either of the treatment methods at a ratio of 1:1 according to a web-based registration program system (based on dynamic allocation by the minimization method). Dynamic allocation by the minimization method is designed to minimize imbalance between treatments taking stratification factors into account. For assignment to prevent large bias. Based on the baseline factors for treatment allocation, including the diameter of tumor, site where the tumor is located, pattern of contrast computed tomography, and institution, an imbalance score is computed. The treatment with the lowest imbalance score is assigned.

The registration secretary will strictly control the program to prevent leaking of the assignment information. Blinding will not be used in this study.

### EUS-FNA procedure

#### Operator

The procedures will be conducted by experienced operators who have performed at least 100 EUS-FNA procedures prior to the initiation of the study.

#### Observation

An EUS device with a convex transducer will be used to observe the target lesions through the lumen of the gastrointestinal tract. In Doppler mode, the EUS device will be used to check whether any large blood vessel crosses the planned puncture route.

#### Puncture

The maximum diameter of the lesion in the direction of the puncture route will be measured in order to determine the position of the puncture needle stopper. Puncture will be performed after retracting the stylet by approximately 5 mm.

#### Strokes

While applying negative pressure using a 10-mL syringe and observing the puncture needle under ultrasound guidance in real time, the needle will be moved back and forth within the lesion approximately 20 times.

#### Processing of specimens

The stylet will be re-inserted into the needle to eject the collected tissues from inside the needle. The specimens will be submitted in a container of formalin.

#### Procedure completion

It is not necessary to use the same needle for subsequent punctures. The operator may freely select the puncture needles for subsequent punctures depending on the size and number of specimens obtained during the first puncture. In this study, only the specimen obtained with the first puncture will be used to evaluate the pre-specified outcomes. The EUS-FNA procedure will be considered complete when the operator determines that sufficient specimens have been collected at the second or subsequent punctures.

### Histopathological diagnosis

Histopathological diagnosis will not be based on cytology but on histology. Specimens fixed with formalin will be embedded in paraffin blocks. Immunostaining and nuclear staining will be performed as needed. In this study, the specimens obtained at the first puncture will be sent to Hoken Kagaku West Japan Co., Ltd, who will prepare the specimens and send them to Kyoto Prefectural University of Medicine for diagnosis. Two experienced pathologists who have performed at least 1000 cytology and histology evaluations of EUS-FNA specimens will assess the specimens independently. The two pathologists will be blinded to the type of needle used. Histology evaluation will be performed to determine: (i) sample cellularity, (ii) preservation of tissue architecture, and (iii) histologic diagnosis. Sample cellularity is classified as rich (≥ 5000 cells), moderate (100–5000 cells), or poor (< 100 cells).

### Final diagnosis

For the purpose of this study, diagnosis is defined as malignant or benign mass. For patients receiving surgical resection, the final diagnosis will correspond to the pathological diagnosis established based on the resected sample. For patients with non-resection, the final diagnosis will be made based on the results of repeat EUS-FNA biopsy, diagnostic imaging such as computed tomography, magnetic resonance imaging, and positron-emission tomography, and observation of the clinical course for at least 1 year. Kamata et al. reported the final diagnosis of 61 patients (27%) was confirmed by surgical resection in 225 patients [10]. So, we expect 30% of patients will receive surgical resection.

### Follow-up period

For the purpose of the present study, all patients will be followed-up for at least 1 year after EUS-FNA.

### Sample size calculation

There have been no reports on the rate of correct diagnosis of pancreatic masses evaluated by EUS-FNA using the 22G Franseen needle. However, a previous study focusing on puncture needles designed to sample sufficient tissue for core biopsy reported that the rate of correct diagnosis was 75% for the conventional needle (EchoTip Ultra needle) compared to 80% for the specially designed EchoTip ProCore needle [10]. Assuming that the rate of correct diagnosis is higher when using the Franseen needle than when using the EchoTip ProCore needle, we expect the rate of correct diagnosis of pancreatic mass malignancy to be 85% when EUS-FNA is performed using the Franseen needle. A sample size of 500 participants (250 per group) would provide a power of 80% (1 − β) considering a two-sided significance level of α = 0.05 (chi-square test) for between-group comparisons of diagnostic rate. Assuming that approximately ten participants per group will have to be considered ineligible for inclusion in the final analysis, the target sample size in this study is established at 520 participants (260 per group).

### Data collection

**Fig. 2:**
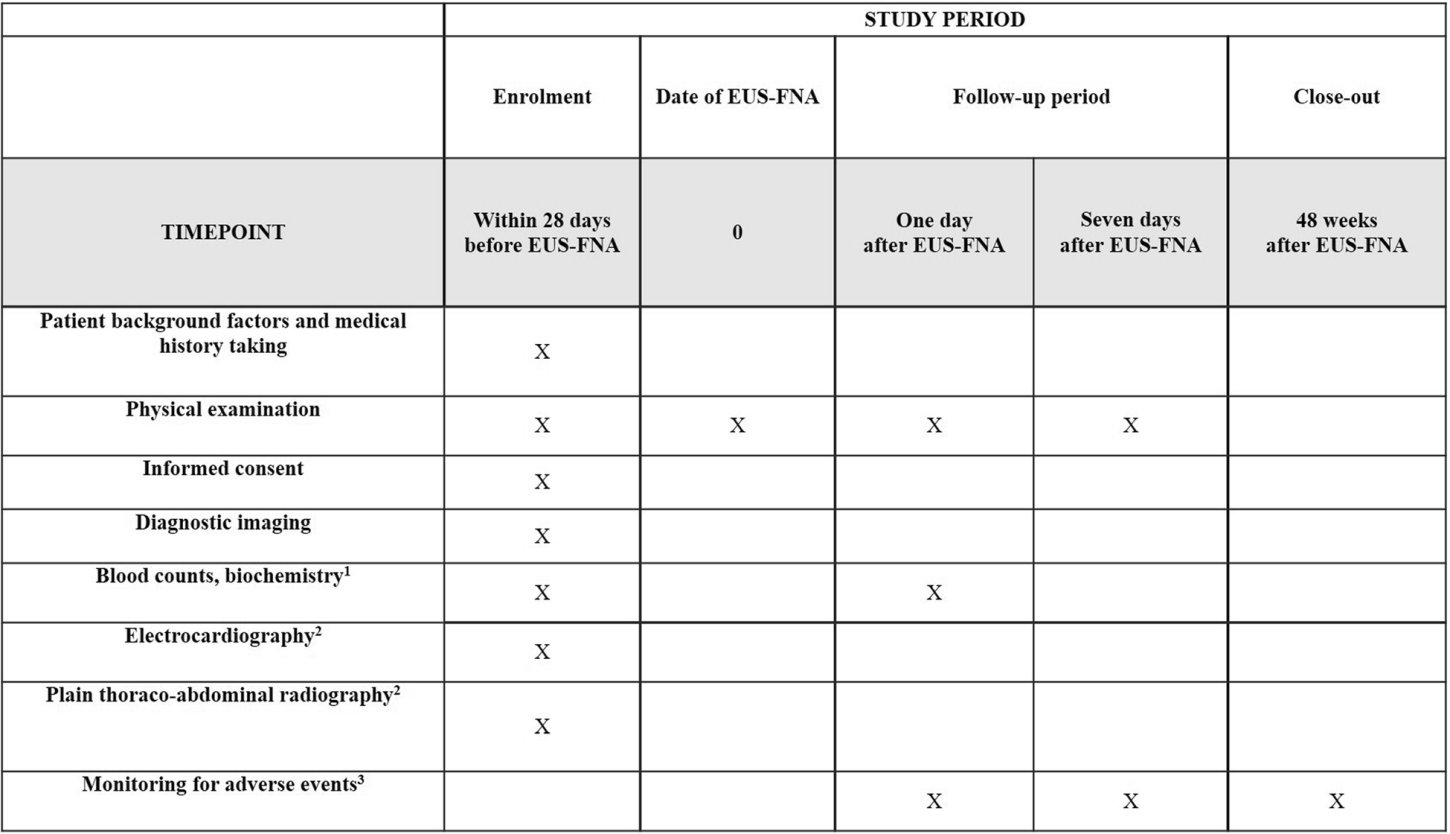
Standard protocol items (SPIRIT): schedule for data collection. ^1^Blood tests to be performed include blood counts, total bilirubin, albumin, alanine aminotransferase, aspartate aminotransferase, γ-glutamyl transpeptidase, alkaline phosphatase, amylase, blood urea nitrogen, creatinine, and C-reactive protein. ^2^Electrocardiography and thoraco-abdominal radiography will be performed for safety evaluation during the pre-treatment observation period. ^3^Adverse events monitored will include all unfavorable events, including adverse reactions not related to the EUS-FNA procedure. *EUS-FNA* endoscopic ultrasound-guided fine-needle aspiration

Data will be collected using a standardized data entry form and entered into the data management system. Figure 2 provides an overview of the types of data collected and the timing of data collection.

### Safety analysis

The severity of adverse events will be graded according to the guidelines issued by the American Society for Gastrointestinal Endoscopy [12]. The incidence rate and proportion of adverse events during follow-up will be calculated for the safety analysis set, both overall and stratified by severity. Clopper-Pearson exact 95% confidence intervals will be estimated for these occurrence proportions.

### Exploratory analysis

Exploratory subgroup analyses will be conducted to investigate the interaction between treatment effects and background factors. In principle, median values will be used for the stratification of the study sample into subgroups according to each factor of interest. Since the subgroup analyses are not statistically powered and no multiplicity adjustment is performed, the results of these analyses will only be interpreted as exploratory. In subgroup analyses, the odds ratio will be used as a summary of therapeutic effects. A Forest plot will be used to summarize the point estimate and 95% confidence interval for each subgroup. The factors and cutoff values to be used for exploratory analysis will be established in a separate statistical analysis plan.

### Monitoring

Visit monitoring will be performed once a year by an independent data monitoring committee. The monitoring committee will collect information on the status of accumulation, inclusion/exclusion criteria, serious adverse events, etc., and strive to provide feedback to participating institutions for early resolution if there are any problems. The monitoring committee will also report the serious adverse events to the committee of efficacy and safety assessment.

## Discussion

The choice of the most suitable needle for the diagnosis of pancreatic masses remains controversial. Several factors may affect the results of EUS-FNA biopsy, including the nature of the target lesion, the experience of the endoscopic expert, the type of needles used, the number of needle passes, and the availability of an on-site cytologist or pathologist. Moreover, specimen processing can vary between institutions, which may be a particularly relevant confounder for the diagnostic ability of EUS-FNA because the specimen collected via needle aspiration is minute. One important advantage of our study is that all samples analyzed are first-pass samples obtained using the same procedure and subjected to histologic analysis in a single facility staffed by experienced pathologists. Furthermore, the study design prevents selection bias because candidates will be recruited from among consecutive patients indicated for EUS-FNA. Finally, the study design prevents information bias because the pathologists will be blinded to the type of needle used.

Some limitations of the present study design should be mentioned. First, a final pathological diagnosis may not be available for all participants (e.g., patients who do not undergo surgery because the mass is deemed to be benign or because of other reasons such as high surgical risk or refusal of surgical treatment). Thus, a minimum of 12 months of clinical follow-up will be conducted to help establish the final diagnosis. To prevent dropout during follow-up, we will carefully monitor the recruitment process, ensure that fully informed consent is obtained, and check that complete registration information is adequately recorded. Second, the study will not consider cytology evaluation (including rapid on-site evaluation). Cytology evaluation is a very important examination that can improve the diagnostic efficacy of EUS-FNA [13]. In the present study, however, we will only evaluate the histology of the whole sample obtained in the first needle pass. The reason for this approach is that the aim of our study is to compare the diagnostic accuracy of EUS-FNA when using different needles, without the added potential benefit of cytology, which may be difficult to quantify. Third, only the results of the samples obtained in the first needle pass will be analyzed. At present, EUS-FNA best practice requires multiple passes [14] because the first pass is insufficient to diagnose pancreatic tumors in some patients. However, to establish a simple set of conditions that allow applying strictly standardized methods in clinical practice, only first-pass data will be evaluated in this study to determine whether the use of Franseen needles rather than conventional needles improves diagnostic accuracy. Results obtained from subsequent passes, in combination with imaging results and follow-up outcomes, will be used to determine the final diagnosis in patients who do not undergo surgery.

This trial will compare the diagnostic accuracy of EUS-FNA biopsy in pancreatic solid lesion samples obtained using conventional needles versus Franseen needles. The outcomes of this study may provide insight into the optimal needle choice for evaluating the malignancy of pancreatic solid lesions, thus aiding in the development of practice guidelines for pancreatic diseases.

### Trial status

Protocol version number: 01, November 26, 2017.

Patient enrollment began on July 17, 2018 and is expected to be completed by November 26, 2019.

## Supplementary information

**Additional file 1.** Standard Protocol Items: Recommendations for Interventional Trials (SPIRIT) 2013 checklist: recommended items to address in a clinical trial protocol and related documents.

## Data Availability

The datasets generated during the study will be available from the corresponding author on reasonable request after termination of data collection.

## Abbreviation

EUS-FNA: Endoscopic ultrasound-guided fine-needle aspiration
